# Regulatory standards and processes for over-the-counter availability of hormonal contraception and drugs for medical abortion in five countries in the Eastern Mediterranean Region

**DOI:** 10.1186/s12961-020-00661-2

**Published:** 2021-04-21

**Authors:** Anne Ammerdorffer, Mark Laws, Arinze Awiligwe, Florence Erb, Wallada Im-Amornphong, A. Metin Gülmezoglu, Lester Chinery, Briana Lucido, Manjulaa Narasimhan

**Affiliations:** 1 Concept Foundation, Geneva, Switzerland; 2 Concept Foundation, Pathumthani, Thailand; 3 Department of Sexual and Reproductive Health and Research, including the UNDP/UNFPA/UNICEF/WHO/World Bank Special Programme of Research, Development and Research Training in Human Reproduction (HRP), World Health Organization, Geneva, Switzerland

**Keywords:** Self-care interventions, Sexual and reproductive health, Contraceptives, Emergency contraception, Medical abortion, Over-the-counter, Prescription-only, Regulatory status, Regulatory process, Eastern Mediterranean Region

## Abstract

The World Health Organization 2019 *WHO consolidated guideline on self-care interventions for health: sexual and reproductive health and rights* includes recommendations on self-administration of injectable contraception, over-the-counter (OTC) oral contraception and self-management of medical abortion. A review of the regulatory status of these two self-care interventions can highlight processes required to ensure that the quality of the medicines and safety of individuals are safeguarded in the introduction and scale-up in countries. This review outlines the legal regulatory status of prescription-only medicine (POM) and OTC contraceptives, including emergency contraception, and drugs for medical abortion in Egypt, Jordan, Lebanon, Morocco and Tunisia using information obtained from internet searches, regulatory information databases and personal contacts. In addition, the review examines whether the national medicines regulatory authorities have documented procedures available to allow for a change in status from a POM to OTC to allow for increased accessibility, availability and uptake of self-care interventions recommended by WHO. Egypt, Jordan and Lebanon have a documented national OTC list available. The only contraceptive product mentioned in the OTC lists across all five countries is ellaOne (ulipristal acetate for emergency contraception), which is publicly registered in Lebanon. None of the five countries has an official documented procedure to apply for the change of POM to OTC. Informal procedures exist, such as the ability to apply to the national medicines regulatory authority for OTC status if the product has OTC status in the original country of manufacture. However, many of these procedures are not officially documented, highlighting the need for establishing sound, affordable and effective regulation of medical products as an important part of health system strengthening. From a public health perspective, it would be advantageous for licensed products to be available OTC. This is particularly the case for settings where the health system is under-resourced or over-stretched due to health emergencies. Readiness of national regulatory guidelines and OTC procedures could lead to increased access, availability and usage of essential self-care interventions for sexual and reproductive health and rights.

## Background

Health care is becoming increasingly people-centred, and individuals are becoming more self-empowered when choosing their health care options, with increasing access to and utilization of information sourced online. The expansion of information platforms and the development of new diagnostics, devices and other digital innovations are changing the interface between individuals and health care workers [1]. The concept of self-care is not new, but the new technologies and interventions have led to an increase in the usage and demand for self-care interventions in all medical fields. Self-care interventions are likely to be part of the solution to the challenge of an estimated shortage of 12.9 million health care workers by 2035 [2].

The World Health Organization (WHO) defines self-care as “the ability of individuals, families and communities to promote health, prevent disease, maintain health, and cope with illness and disability with or without the support of a health-care provider” [1]. Self-care interventions recommended by WHO are evidence-based and can include information about a sexual or reproductive health issue as well as ways in which individuals can obtain drugs, devices, diagnostics and/or digital products fully or partially separate from formal health services, and which can be used with or without the direct supervision of a health worker [3]. In the field of sexual and reproductive health and rights (SRHR), several self-care interventions are available, including self-sampling for human papillomavirus (HPV) and sexually transmitted infections, and contraceptives for self-administration [1]. Self-care interventions may play an important role in supporting efforts to reach the United Nations Sustainable Development Goal (SDG) 3 and 5 (target 3.7 and target 5.6), to ensure universal access to sexual and reproductive health (SRH) care services by 2030 [4]. Self-care interventions are particularly important for SRHR, since they can empower individuals to engage in informed decision-making regarding their contraceptive use and fertility intentions.

Despite efforts to scale up modern contraceptive use, many countries are still struggling with high unmet need for contraception. Among the 1.9 billion women of reproductive age (15–49 years), 190 million women want to avoid pregnancy and do not use any contraceptive method [5]. In the WHO Eastern Mediterranean Region (EMR), the overall prevalence of contraceptive use is 48%, and the unmet need for contraception is the second highest among all WHO regions [6]. In Egypt, Jordan, Lebanon, Morocco and Tunisia, the unmet need is 12%, 12%, 13%, 14% and 7%, respectively [6]. Common factors contributing to the high unmet need in these countries and other lower-middle-income and low-income countries include low access to SRH services; inadequate quality of contraceptive care; poor family planning counselling; poor awareness of family planning benefits, existing services and types; misconceptions related to health and religious teachings; lack of social and spousal support; and sociocultural barriers and unsupportive environment. In addition, cultural views on family size and religious beliefs can play a role in the acceptance and usage of contraception in EMR countries [6]. In 2019 and 2020, several consultative meetings on adopting self-care interventions for SRHR in the EMR were organized by the WHO Regional Office for the Eastern Mediterranean. The purpose of the meetings was to ensure the adoption and implementation of self-care interventions for SRH including OTC contraception and subcutaneous depot medroxyprogesterone acetate injection (DMPA-SC), and ensure their integration into national health policies, regulations, programmes and services [7, 8]. The availability of self-administered injectable DMPA, over-the-counter (OTC) provision of oral contraceptive pills and self-management of abortion to increase modern contraceptive use were among the topics discussed. The meeting led to multiple considerations for the EMR countries present regarding the introduction and scale-up of self-care interventions for SRHR. This included initiating policy, regulation, programmatic and service delivery considerations for self-care interventions for SRHR [7], as well as considering the countries’ specificities to ensure adoption.

The rise in demand and uptake of self-care interventions for contraception and medical abortion have direct implications on the regulatory status of these products and the processes required to ensure that the quality of the medicines and safety of individuals are safeguarded. Specifically, the necessary procedures to allow for a change in status from a prescription-only medicine (POM) to OTC provision need to be overseen and regulated by the national medicines regulatory authorities (NMRAs) to ensure that the manufacture, procurement, distribution and especially the use of medicines are effectively regulated and documented. Medicines suitable for use as a self-care intervention should continue to be assessed in order to ensure their safety and efficacy, and the labelling of medicines and client information instructions should be understandable for users without additional explanation from health care workers. In addition, it is important that the user instructions contain clear information on how to request support and further information about the product, if needed. If the self-care intervention is ineffective or poses a risk to the user, or if instructions for its use are unclear, it could lead to therapeutic failure and also reduce confidence and trust in the overall health system [9]. It is essential that NMRAs have standardized, clear and rigorous regulatory processes when making SRH medicines available without prescription as a self-care intervention and, more importantly, prevent adverse or unintended health outcomes.

We aimed to provide an overview of existing self-care interventions for contraceptive and medical abortion products and to document the regulatory and other formal statutory processes and pathways that allow for self-administration of these target products, to the extent that they exist, in five EMR countries (Egypt, Jordan, Lebanon, Morocco and Tunisia).

## Methods

### Definition of contraceptives and medical abortion

Hormonal contraceptive methods are traditionally and formally designated as prescription medicines in most countries. Types of hormonal contraceptives included in this review in the five selected countries include oral pills (combination pills and/or progestin-only pills) of different formulations, emergency contraceptives, injectables, and transdermal (patches) and vaginal products (vaginal ring). Certain contraceptives are not included in the review, as they are provider-dependent (subdermal implants, intrauterine devices, and surgical interventions), are less commonly used (i.e. diaphragm and cervical cap), or are not considered as medicines and are currently widely available for purchase without prescription (such as male condoms).

Drugs for medical abortion, a procedure that uses medication to end a pregnancy, include both misoprostol (for misoprostol-only interventions) and the WHO-recommended regimen, a combination of mifepristone and misoprostol. Self-administered contraception and self-management of medical abortion are both recommended by WHO.

In this review, we focused primarily on stand-alone OTC interventions, for which no prescription or consultation is needed before purchase and use—either formally or informally. However, to provide a more comprehensive overview of the interventions in the field of contraceptives and medical abortion, self-care interventions for which a single initial prescription is needed are also included, as in some countries they could be used as stand-alone OTC interventions as well.

### Search strategy

We developed a data collection form that included questions around the availability of these medicines and the national procedures in place for registering as a prescription-only and OTC medicine, as well as the process and procedure for a change of status from one to the other. Our primary search strategy included internet-based searches of both international and national websites. For missing data, we interviewed and/or contacted experts in the field of SRHR residing in and familiar with the national regulatory contexts.

### Data sources

#### General websites and information

Regulatory information was obtained primarily from the Cortellis Regulatory Intelligence™ database, which provides summaries of the existing regulatory processes and procedures applicable to the countries investigated.

Information on the availability of emergency contraception in each country was taken from the International Consortium for Emergency Contraception’s (ICEC) website [10]. National essential medicines lists mentioned on the WHO regional EMR website were also examined [11]. In the online national drug databases, we used the Anatomical Therapeutic Chemical (ATC) classification. We identified the products in sector G (genito-urinary system and sex hormones), specifically G02AD (prostaglandins, including misoprostol), G02B (contraceptives for topical use), G03A (hormonal contraceptives for systemic use, including G03AD emergency contraceptives) and G03XB (progesterone receptor modulators, including mifepristone and ulipristal). If the ATC classification was not available, we used the keywords ethinylestradiol, levonorgestrel, misoprostol, mifepristone, medroxyprogesterone and ulipristal acetate, and combinations, derivatives and synonyms. PubMed and local news items (via search engines) were used to find background information on contraceptive usage, regulatory processes, and OTC availability and usage. We checked the WHO Global Abortion Policies Database to confirm and cross-check [12].

In addition to online-accessible information, we reached out to local experts (see acknowledgements) in the different countries and a European pharmaceutical company that has registered target medicines in EMR countries, for their experience with regulatory procedures, requirements and processes for contraceptives. A template questionnaire was used to obtain information from the local experts. The questions focused on (1) the availability of a national guideline for contraceptive self-care interventions, (2) the availability of formal regulatory procedures to switch or reclassify from POM to OTC, (3) known historical reclassifications from POM to OTC in the country and which government agencies where involved, and (4) the current practice/reality for obtaining contraceptives and medical abortion products from a pharmacy.

#### Country-specific information

The WHO EMR includes 21 Member States, of which five countries (Egypt, Jordan, Lebanon, Morocco and Tunisia) were selected for the study [13]. The criteria for the selection was known ongoing discussions on OTC availability of contraceptives, and the anticipation of having access to relevant information within the time frame of the project. In addition, all five countries joined WHO consultative meetings in 2019 on adopting the guideline on self-care interventions for SRHR in the EMR, demonstrating their interest in the topic [7, 14].

Egypt: The website from the Egyptian Ministry of Health contains information on registered medicines and regulatory processes. However, the website was not accessible outside Egypt.
Jordan: The database for drugs provided for registration can be found on the website of the Jordan Food and Drug Administration (JFDA). In the Arabic search engine, searches can be made on ATC classification, and the results include English translations. In addition, we used the Jordan Model List of Essential Medicines, 1st Edition [15], and the Jordan OTC spreadsheet which lists medicines that are issued without a prescription approved as of May 2018 [16]. Regulatory information can be found on the website as well [17].
Lebanon: We made use of the Lebanon National Drug Index, Fifth Edition, 2015 document and the online Lebanon National Drugs Database [18]. OTC products are listed in the National OTC Medicines List—First Edition 2018 [19]. The website of the Ministry of Public Health (MoPH) contains regulatory information.
Morocco: We used the information included in the website from the Directorate of Medicines and Pharmacy [20].
Tunisia: The online database for medicines from the Department of Pharmacy and Medicines in Tunisia (in French) can be classified by therapeutic class, including sex hormones and modulators of the genital system [21]. Regulatory information can be found on the Department of Pharmacy and Medicines website as well.

We added new information or validated the information collected through our contacts with local experts in Egypt, Lebanon, Jordan and Morocco.

## Results

### Egypt

The Egyptian Drug Authority (EDA) is the regulatory body, affiliated with the Ministry of Health, with authority over the pharmaceutical industry. It regulates the safety and quality of human and veterinary medicines, biologicals, medical devices, cosmetics, dietary supplements and pesticides. Marketing authorization is not allocated to any product unless the necessary certificate of compliance is obtained from the National Organization for Drug Control and Research (NODCAR) within the EDA.

In 2014, the Ministerial Decree No. 442 (non-prescription medicines list) specified the list of 127 OTC products permitted to be dispensed by pharmacies without prescription. Prior to this Ministerial Decree, no OTC status was available in Egypt, and the only legal status change permitted was between a medicinal product and a food supplement [22]. From practical experience, a switch from prescription medicine to food supplement or vice versa is permitted if its relevant status has changed in the country of origin or in a reference text (the British National Formulary, United States Pharmacopeia and European Pharmacopoeia). The change can be initiated by the marketing authorization holder (MAH) or requested by the EDA and then reviewed by the registration department. Once approved, a new certificate with product status is issued. No timeline is specified. However, there is no official documented procedure in Egypt to change the status of a product from POM to OTC, and therefore no mechanism for requests to add products to the official OTC list.

According to the rules and regulations, combined oral contraceptives and progestin-only pills are supplied by trained health providers. However, in general, the prevailing practice is for community pharmacies to provide hormonal contraceptives without a prescription. Abortion is legally possible only in cases of risk to the mother’s life or major fetal anomalies, and therefore access to drugs for medical abortion is highly restricted and controlled [23]. Misoprostol, which is formally indicated and registered as an anti-ulcer medication, is only allowed at hospitals for its use as a uterotonic (personal communication).

### Jordan

The JFDA is the pharmaceutical regulatory body in Jordan. It was created in 2003 as the sole national competent authority for drug safety and efficacy and food safety and quality.

There is no documented process for a change of status from POM to OTC product or no specific variation type for changing a legal status. In practice, the MAH or local representatives can submit a request for a legal status change of their registered product to the JFDA Registration Division, providing justification for the proposed amendment. For example, if the product is approved as OTC in the country of origin. It should be noted that in any case, a product can only be approved as OTC in Jordan if it is also an OTC in its country of origin [24].

JFDA has compiled a list of medicines that are issued without a prescription (latest update May 2018) and that are subject to the principles of drug registration and pricing bases [16]. In 2019, the JFDA issued a notice (Circular 2/1/1/8135) inviting drug suppliers to review the prescription status of their OTC products (JFDA OTC list) and apply to update the way their products are provided in terms of status [25]. This notice required the distributors to make their applications within 3 months from the date of the circular [25] and to include a letter from the responsible person and from the company and the prescription status of the originator/reference drug.

In Jordan, progestin-only pills, combined oral contraceptive pills and injectable contraceptives are available in the country. However, no hormonal contraceptives, emergency contraception or medical abortion drugs are included in the national OTC list.

### Lebanon

The MoPH contains three directorates, of which the Directorate of Medical Care is responsible for pharmaceutical regulation.

According to two laws (Article 43 of Law No. 367 from 1994 and Articles 46 and 47 of Law No. 91 in 2010), pharmacists do not have the right to dispense any medicine that is not requested through a prescription [26]. The exception is when the medicine is mentioned in a list established by the Orders of Pharmacists and Physicians. In 2018, the first edition of this national OTC list was released, which was compiled based upon the ATC classification system.

There is no official procedure in Lebanon to change the status of a product from POM to OTC. Practical experience shows an informal procedure for the addition of products to the OTC medicines list. We consider that the information likely required by the MoPH to support a status change would include (i) evidence of legal (OTC) status in a designated stringent regulatory authority (SRA) country and/or country of origin, (ii) clinical and safety data to support the change, and (iii) the latest published literature and/or any other update of the product information related to the legal status change request.

In Lebanon, combined oral contraceptives, progestin-only pills, injectable contraceptives (Depo-Provera) and two formulations of emergency contraceptives (ulipristal acetate and levonorgestrel) are available. Medical abortion products (misoprostol only) are sold with a prescription with or without provider consultation and with or without on-site counselling and administration.

In the National OTC Medicines List, ellaOne (ulipristal acetate, 30 mg) is present, but no other contraceptives, emergency contraception or medical abortion drugs are listed. ellaOne is not provided by the MoPH but is available for purchase from pharmacies.

### Morocco

The pharmaceutical regulatory body of Morocco is the Directorate of Medicines and Pharmacy (La Direction du Médicament et de la Pharmacie, DMP). Established in 1994, it is one of the eight central governmental departments of the Ministry of Health.

There is no formal procedure for the switch of a POM to an OTC product, and there is no guidance available for submission of a variation to make the change [27].

In practice, the MAH can make requests to the DMP to change the legal status of their product. The request relies on a formal change of the legal status of the product in the country of origin. It is highly unlikely that a product in Morocco can have OTC status if this is not the case in the original country of manufacture. From our review, it appears that the DMP would require the following information to support a change to OTC: (i) the evidence of legal (OTC) status in an SRA country and/or country of origin and (ii) the clinical, safety and pharmacovigilance data to support the change. After submission, DMP indicates that it will communicate its final decision in writing within 2–4 weeks. A new registration certificate with the new registration status would then be issued. The formalization of this procedure and issuance of guidance around its use would provide reassurance to MAHs considering a change in product status that requests would be seriously considered by the DMP using standardized and transparent criteria.

In Morocco, progestin-only pills, combined oral contraceptives, vaginal rings and two emergency contraceptive formulations (ulipristal acetate and levonorgestrel) are available. Misoprostol is included in the essential medicines list as an “oxytocic”. Abortion is only allowed when the mother’s life is at risk, and misoprostol can be used for abortion or post-abortion care in a hospital setting [23].

### Tunisia

The Department of Pharmacy and Medicines (La Direction de la Pharmacie et du Médicament, DPM) is the technical-administrative unit of the MoPH. It manages all administrative aspects related to pharmacy, medication and related activities.

There is no formal procedure for the switch of a POM to an OTC product, and there is no guidance available for submission of a variation to make the change. However, an unofficial process is outlined wherein the MAH makes an application to the DPM for a change in the status of a prescription-only medicinal product to an OTC medicinal product in Tunisia. The DPM requires the following in support of the application: (i) switch of drug status in other countries (worldwide product status), (ii) clinical and preclinical data supporting the change, (iii) how long the product has been marketed in Tunisia (and other countries) and (iv) the history of the safety profile of the product.

There is no published list of medicines approved for OTC supply in Tunisia. A classification of OTC products exists, and is determined during the initial registration of the product. In Tunisia, OTC products are referred to as “produits conseil” (advice products) and may differ slightly from the typical OTC classification used in European countries. The two differences identified are that the medicinal product is not directly accessible to the individual and must be delivered by the pharmacy staff under the pharmacist's responsibility, and that the price of the product is defined by the Ministry of Health [28].

In Tunisia, combined oral contraceptives, progestin-only pills and emergency contraceptives (ulipristal acetate and levonorgestrel) are registered. Mifepristone is registered both alone and as a combination pack with misoprostol for medical abortion.

An overview of the regulatory processes in the five selected countries in the EMR is shown in Table 1.

**Table 1:** Overview of regulatory processes in five selected countries in the EMR

	Formal procedure for the switch from POM to OTC product available	Online database of registered medicines	Actual practice for the switch from POM to OTC	National OTC list available (year available)	Contraceptives included in OTC list
Egypt	No	Yes (not accessible outside Egypt)	Medicinal product to food supplement	Yes (2014)	No
Jordan	No	Yes (in Arabic, ATC classification)	OTC in the country of origin	Yes (2018)	No
Lebanon	No	Yes	Informal procedure, OTC in the country of origin	Yes (2018)	Yes (ellaOne)
Morocco	No	Yes	OTC in the country of origin	No	N/A
Tunisia	No	Yes (classified by therapeutic class)	Application for change of status	No	N/A

*POM* prescription-only medicine, *OTC* over the counter, *N/A* not applicable

## Discussion

The main finding of our review is the lack of clear guidance on OTC registration and transition from POM to OTC for hormonal contraception and medicines for medical abortion across five selected countries in the EMR. While hormonal contraception seems to be available through community pharmacies, regulatory norms are often missing. The availability of medical abortion drugs is legally restricted across all five countries.

We identified that all five countries have well-structured departments within their ministries of health responsible for the safety and regulation of pharmaceuticals. On all the official websites, an online database is available which includes the national registered medicines, and depending on the website, searches can be made by product name, active ingredient, ATC classification or thematic subject such as gynaecology products. To our knowledge, based upon this review, Egypt, Jordan and Lebanon have a documented national OTC list available. The only contraceptive product mentioned in OTC lists across all five countries is in Lebanon for ellaOne (ulipristal acetate for emergency contraception). None of the other reviewed methods of contraception (oral, vaginal or transdermal), emergency contraception or drugs for medical abortion are included in the national OTC lists for Egypt, Jordan, Tunisia or Morocco. In these countries, which have a definitive listing of products legally classified as OTC, it can therefore be assumed that all registered products not included in the OTC list, by law, require a prescription from a health care worker.

A strong recommendation from the *WHO consolidated guideline on self-care interventions for health: sexual and reproductive health and rights* (recommendation 10) states that self-administered injectable contraception should be made available as an additional approach to deliver injectable contraception for individuals of reproductive age [29]. We identified that the intramuscular injectable contraceptive Depo-Provera is available in Egypt, Jordan and Lebanon. In Morocco, there is an ongoing pilot introduction project for the self-administration of DMPA-SC to prepare for its introduction at the national level [30] (personal communication). According to the registration list (brand name Sayana Press manufactured by Pfizer), it is registered in Egypt, Jordan and Morocco, with self-administration approved in the both Jordan and Morocco [31]. We did not find evidence of registered injectable contraceptives in Tunisia, though the estimated prevalence of contraceptive use among women of reproductive age (15–49 years) includes injectables at 0.6%. In comparison, this percentage is 6.2, 0.5, 0.1 and 1.3% for Egypt, Jordan, Lebanon and Morocco, respectively [5]. Self-administration is a step toward making contraceptives more accessible for people. However, there is at present insufficient information on the OTC availability of these products.

The OTC availability of oral contraceptive pills (not including emergency contraception) is also strongly recommended by WHO [29]. In all five countries, a broad range of oral contraceptives (progestin only and combined pills) are registered and available. However, none of these products are mentioned in the national OTC lists for Egypt, Jordan or Lebanon, and we could not find documented evidence that they are available OTC in Morocco and Tunisia. It should be noted that it is possible that the updated national OTC lists may not be publicly available, in particular the 2014 document from Egypt. However, other sources do mention that oral contraceptives are available as OTC [32]. Oral contraceptives are formally available as OTC without screening in Egypt, formally available as OTC with screening in Tunisia, and informally available OTC in Morocco and Jordan. This information was obtained via personal contact with in-country contacts, but has not been officially documented by the national health authorities. Further research and the establishment of normative guidelines is needed in these settings to clarify and improve the policies for the designation of OTC status products from a regulatory and statutory perspective.

While none of the five countries has an officially documented procedure to apply for the change from prescription-only to OTC, in practice all the countries have some form of application process. In Jordan, Lebanon and Morocco, the legal status of the product in the country of origin plays an important role. In cases where the product is not legally available OTC in the original country of manufacture, there is no or only a very limited chance that the product can transition to OTC. On the other hand, if a product is legally registered as OTC in the original country of manufacture, the process to apply for OTC status in Jordan, Lebanon and Morocco seems relatively simple. In the OTC list of Jordan, “approved as OTC in the country of origin (i.e. France)” is mentioned several times in the additional information column. The manufacturer of ellaOne informed us that once the company obtained OTC status in the European Union, they informed the ministries of health, who switched the registration status to OTC. Open questions remain as to whether it is possible to request an OTC change following OTC designation in the country of origin, whether there is no market for OTC contraceptives (not supported by the evidence), whether national authorities are not in favour of accepting contraceptives as OTC or, perhaps, whether the existing informal procedure for providing OTC is considered sufficient. From a regulatory perspective, ellaOne could be OTC in both Lebanon and Jordan. However, the fact that ellaOne is described as OTC in Lebanon but not in Jordan shows that the approval of (emergency) contraceptives as OTC can be a complex process due to the above factors or other potential cultural influences.

We assessed the formal regulatory status of selected reproductive health products in the EMR, and examined the regulatory processes allowing for these products to be switched from POM to OTC. In doing so, we were able to demonstrate a lack of structured, well-documented and accessible guidelines and/or procedures in these countries necessary to make the switch from POM to OTC. One limitation of the study is the accessibility of original information and resources. While some websites of the ministries of health contained detailed information, others were not accessible outside their country, which limited our search and also prevents foreign MAHs from accessing information on possible application for OTC product status. Except for Tunisia, the identified information and recourses were validated by local experts. Secondly, it is important to mention the large difference between the official regulations on hormonal contraceptives and medical abortion drugs versus the actual availability and usage in real life. Hormonal contraceptives are often available at pharmacies without prescription (either with or without a screening), and emergency contraceptives and medical abortion drugs can be accessed on the local black market (personal communication). Simple internet searches in each country also identified (online) pharmacies where products could be bought, and advice is given on how to use general hormonal contraceptives as an alternative method of emergency contraception. Having to resort to illegal alternatives as the only means of accessing (emergency) contraception and medical abortion drugs is a situation that needs to be avoided, as products obtained in this manner could be substandard, of low quality, and unsafe and/or ineffective, and thereby could lead to adverse and unintended health outcomes. The ability to obtain OTC contraceptives and emergency contraceptives gives people additional choice, and when the information associated with these OTC products is correct and appropriate, this can lead to better-informed health decision-making and improved health outcomes for individuals, their families and their communities.

For many NMRAs, self-care interventions are new products, although several of these have been available for some time, including condoms. While it is important for NMRAs in the developing world to be aware of international processes and recommendations, such as those from the European Medicines Agency (EMA), it is equally important that they are guided primarily by evidence considered in the light of local risk–benefit analysis. The capacity for countries to do so can be enhanced by regional collaboration, resource-sharing and training. WHO can support these processes by providing independent scientific and technical advice. The provision of quality SRH services within health facilities can be complemented with self-care interventions that are available OTC, with appropriate safeguards in relation to confidentiality and supporting people’s autonomy. The NMRAs can grant OTC availability of self-care interventions as part of their pharmacovigilance, through a programme of surveillance which can assess issues including regulatory requirements, controlling risk and evaluating public health impact. Labelling and package inserts will also need to be monitored to ensure that they present appropriate messages for home use of self-care interventions. While acknowledging the large influence culture and tradition can have on the transition of SRH products to OTC status in EMR countries, the availability of guidelines and procedures for formally designating these products as OTC could lead to profound changes in the field and increased access, usage and uptake of contraceptives and medical abortion drugs among people in the region.

## Conclusion

Public confidence in health products depends on confidence in the integrity of regulatory oversight. From a public health perspective, it would be advantageous for licensed products to be available OTC. This is particularly the case for settings where health systems may be under-resourced or over-stretched due to health emergencies. In the five EMR countries where the legal regulatory status of POM and OTC contraceptives, including emergency contraception and drugs for medical abortion, were reviewed, self-care interventions for hormonal contraceptives is widespread, and is dependent upon the in-country registration and availability of each product. Except in Tunisia, access to medical abortion drugs is highly restricted.

We conclude that in the selected countries in EMR there is a need to establish sound, affordable and effective regulation of medical products as an important part of health system strengthening, including for designating a licensed product as OTC and a transfer of status from POM to OTC in order to formalize existing self-care interventions of non-provider-dependent hormonal contraceptives and, where legally permitted, medical abortion drugs, under the auspices of the NMRAs. In doing so, the national regulatory systems can remain relevant, current and flexible as technology evolves, with additional self-care interventions to support increased choice for individuals and health systems. Reviewing situations in other geographical regions would provide a deeper understanding of self-care interventions for SRHR as nationally approved and regulated OTC products.

## Data Availability

Not applicable; all data are discussed in the manuscript.

## Abbreviations

DMP: Direction du Médicament et de la Pharmacie
DMPA: Depot medroxyprogesterone acetate
DPM: Direction de la Pharmacie et du Médicament
EDA: Egyptian Drug Authority
EMA: European Medicines Agency
EMR: Eastern Mediterranean Region
JFDA: Jordan Food and Drug Administration
MAH: Marketing authorization holder
MoPH: Ministry of Public Health
N/A: Not applicable
NMRA: National medicines regulatory authority
NODCAR: National Organization for Drug Control and Research
OTC: Over the counter
POM: Prescription-only medicine
SC: Subcutaneous
SRH: Sexual and reproductive health
SRHR: Sexual and reproductive health and rights
WHO: World Health Organization
